# Direct Comparison Between the Addition of Pembrolizumab or Bevacizumab for Chemotherapy-Based First-Line Treatment of Advanced Non-Squamous Non-Small Cell Lung Cancer Lacking Driver Mutations

**DOI:** 10.3389/fonc.2021.752545

**Published:** 2021-09-29

**Authors:** Jiatao Liao, Chang Liu, Qianqian Long, Xianghua Wu, Huijie Wang, Hui Yu, Si Sun, Yao Zhang, Ying Lin, Xinmin Zhao, Jialei Wang

**Affiliations:** ^1^ Department of Medical Oncology, Fudan University Shanghai Cancer Center, Shanghai, China; ^2^ Department of Oncology, Shanghai Medical College of Fudan University, Shanghai, China

**Keywords:** non-small-cell lung cancer, pembrolizumab, bevacizumab, pemetrexed, biomarker, first-line treatment

## Abstract

**Background:**

The addition of bevacizumab or pembrolizumab to pemetrexed-platinum chemotherapy has produced significant clinical benefits to patients with untreated, advanced non-squamous non-small cell lung cancer (NSCLC) lacking targetable genetic aberrations. However, the direct comparison between these two first-line treatments needs to be investigated.

**Methods:**

We retrospectively investigated the medical records of 102 patients with stage IIIB~IV non-squamous NSCLC, and without sensitizing EGFR/ALK/ROS1 alterations. All patients received pembrolizumab or bevacizumab plus pemetrexed-platinum chemotherapy as the first-line treatment between December 2018 to April 2021 at Fudan University Shanghai Cancer Center. Assessments included progression-free survival (PFS), overall survival (OS), objective response rate (ORR), disease control rate (DCR), and adverse events (AEs). We also evaluated the prognostic biomarkers in the overall population and explored potential predictive biomarkers to aid the selection of optimal treatment regimens.

**Results:**

The median PFS was 10.0 months in the pembrolizumab group and 9.2 months in bevacizumab group (HR = 1.006; *P* = 0.982), while the median OS was not reached in either group (HR= 1.193; *P* =0.714). ORR was 36.7% versus 43.4% (*P* = 0.548) and DCR was 89.8% versus 92.5% (*P* = 0.735) in the pembrolizumab and bevacizumab groups, respectively. In the overall study population, baseline lymphocyte to monocyte ratio (LMR) >1.95 (HR = 0.312, P < 0.001) was an indicator of longer PFS. The presence of baseline bone metastasis (HR = 4.107, *P* = 0.009), baseline lactate dehydrogenase (LDH) >300 U/L (HR = 4.300, *P* = 0.025) and LMR ≤1.95 (HR = 5.291, *P* = 0.039) were associated with inferior OS. Baseline neutrophil-to-lymphocyte ratio (NLR) ≤3.10 was predictive of significantly favorable OS in the bevacizumab combination treatment (HR = 5.073, *P* = 0.039). The safety profiles were generally comparable between the two groups.

**Conclusions:**

In patients with chemotherapy-naive, advanced, non-squamous NSCLC who lack driver mutations, the efficacy and safety of pembrolizumab and bevacizumab when combined with pemetrexed-platinum were comparable. For patients with baseline NLR ≤3.10, the bevacizumab combination therapy elicited significantly better OS benefits.

## Introduction

Despite the benefits of obtaining early diagnoses and interventions, approximately 57% of lung cancer patients are not diagnosed until the presence of metastatic disease. The result of such untimely diagnoses is a low 5-year survival rate of only 5% (1). For advanced non-squamous non-small cell lung cancer (NSCLC) patients who lack actionable oncogenic drivers and good performance status (PS), the standard front-line treatment is platinum-doublet chemotherapy (2).

Other treatment regimens, e.g., angiogenesis inhibitors and immune checkpoint inhibitors in combination with the standard chemotherapy, have been developed for first-line standard treatment and provided better efficacy comparing to chemotherapy alone. Bevacizumab, an antibody against vascular endothelial growth factor (VEGF), has been proved to induce regression of new blood vessels and facilitate the delivery of cytotoxic drugs to the tumors (3). The pivotal ECOG4599 trial is the first phase III study to demonstrate that the addition of bevacizumab to first-line standard chemotherapy in advanced non-squamous NSCLC patients can extend the overall survival (OS) to more than 1 year (4). The phase III BEYOND study was also performed to confirm those findings in a Chinese patient population and showed an improvement in median OS from 17.7 to 24.3 months in the bevacizumab plus platinum-doublet chemotherapy group compared to patients receiving chemotherapy alone (5). Pembrolizumab, a monoclonal antibody that inhibits programmed death-1 (PD-1) and modulates immune response, has been shown to provide significant survival benefits when administered as monotherapy in patients harboring a programmed death-ligand 1 (PD-L1) tumor proportion score (TPS) of more than 50% (6), or when administered in combination with platinum-based chemotherapy regardless of PD-L1 expression (7, 8). According to the KEYNOTE-189 trial, pembrolizumab plus pemetrexed and platinum has manifested significantly improved median progression-free survival (PFS, 9.0 *vs.* 4.9 months; hazard ratio [HR] = 0.49) and median OS (22.0 *vs* 10.6 months; HR = 0.56) with manageable toxicity in untreated, metastatic non-squamous NSCLC patients without targetable mutations (9). Despite their benefits to patients, it is still elusive about the selection criteria between bevacizumab and pembrolizumab. In fact, no direct comparison on the survival benefits between pembrolizumab or bevacizumab combined with chemotherapy has been completely reported.

A major barrier to optimal treatment selection is the lack of robust biomarkers. For instance, the prevailing biomarkers such as PD-L1 and tumor mutational burden (TMB) have limitations in stratifying patients and maximizing the clinical benefits (10). This partially contributes to the observation that 15% to 20% of NSCLC patients cannot obtain benefits from immunotherapy (11). As inflammation plays an important role in tumor growth and progression (12), the peripheral hematological markers can function as another class of useful biomarkers. Indeed, lactate dehydrogenase (LDH), neutrophil-to-lymphocyte ratio (NLR), derived neutrophil-to-lymphocyte ratio (dNLR, neutrophil count/[leukocyte count-neutrophil count]), platelet-to-lymphocyte ratio (PLR), lymphocyte-to-monocyte ratio (LMR) and absolute eosinophil count (AEC) have been reported as prognostic immune-based biomarkers in patients with stage IV NSCLC, as well as in NSCLC patients receiving immunotherapy (13–17). Being readily available, low-cost, and minimally invasive, these blood indicators thus have great potential in prognosis prediction and treatment selection of patients with NSCLC.

In this study, we compared the first-line treatments of using pembrolizumab-pemetrexed-platinum combination versus bevacizumab-pemetrexed-platinum combination in advanced non-squamous NSCLC without sensitizing EGFR/ALK/ROS1 mutations. Based on the studied population of patients, our biomarker analyses further identified prognostic and predictive markers that can potentially differentiate NSCLC patients and promote the clinical practice.

## Material and Methods

### Patients

In this retrospective cohort study, we screened the medical records of 162 patients who received ≥2 courses of first-line pemetrexed-platinum with pembrolizumab or bevacizumab treatments between December 2018 and April 2021 at Fudan University Shanghai Cancer Center. Patient enrollment criteria included (1) patients were pathologically confirmed as stage IIIB~IV non-squamous NSCLC lacking EGFR/ALK/ROS1 driver alterations; (2) patients were treatment-naive; (3) baseline Eastern Cooperative Oncology Group (ECOG) performance-status (PS) score of 0 or 1; and (4) complete medical records. A total of 102 eligible patients were finally included in the study. Data were cut off at the last follow-up on April 21, 2021.

This study was approved by the Institutional Review Board of the Fudan University Shanghai Cancer Center, and was performed in compliance with the Helsinki declaration.

### Treatments

In the pembrolizumab group, patients intravenously received pembrolizumab (200 mg) plus pemetrexed (500 mg/m^2^) and cisplatin (75 mg/m^2^) or carboplatin [area under the concentration-time curve (AUC), 5 mg/mL per min] every 21 days for 4 or 6 cycles. Pembrolizumab with or without pemetrexed was used subsequently as maintenance therapy every 21 days until the occurrence of disease progression or unmanageable toxic effects.

In the bevacizumab group, patients intravenously received bevacizumab (7.5 mg/kg) plus pemetrexed (500 mg/m^2^) and cisplatin (75 mg/m^2^) or carboplatin (AUC, 5mg/mL per min) every 21 days for 4 or 6 cycles. Bevacizumab with or without pemetrexed was used subsequently as maintenance therapy every 21 days until the occurrence of disease progression or unmanageable toxic effects.

### Assessments

The tumor response was evaluated every two cycles of therapy according to the Response Evaluation Criteria in Solid Tumors (RECIST) version 1.1.

Considering the FDA’s guidance on *Clinical Trial Endpoints for the Approval of Cancer Drugs and Biologics* (December 2018), we defined PFS as the date from the initiation of treatment until the occurrence of progressive disease (PD) or death from any cause (whichever occurred first). OS was defined as the date from the initiation of treatment until the date of death from any cause.

The objective response rate (ORR) was defined as the proportion of patients with a complete response (CR) or partial response (PR) as the best response. The disease control rate (DCR) was defined as the proportion of patients with CR, PR, or stable disease (SD) as the best response.

Adverse events (AEs) were assessed using the National Cancer Institute Common Terminology Criteria for Adverse Events (CTCAE) version 5.0.

### Statistical Analysis

Demographic characteristics were summarized using frequency and percentage for categorical variables and median and interquartile range (IQR) for continuous variables. Differences in baseline clinicopathologic characteristics, ORR, and DCR between the groups were assessed using chi-square or Fisher’s exact test. Differences in age and baseline hematologic parameters were assessed using the Mann-Whitney U test. Survival was analyzed using the Kaplan-Meier method and log-rank test. AEs were summarized using percentages and frequency counts.

To investigate the prognostic biomarkers, univariate and multivariate analyses were conducted to explore the association between clinicopathological features and PFS or OS using Cox proportional hazard regression. The optimal cutoff values for hematologic parameters were assessed using X-Tile (18). Variables with a *P* < 0.10 in the univariate analyses were included in multivariate analyses. The data were shown as hazard ratios (HRs) and 95% confidence intervals (CIs).

To identify the predictive biomarkers, a treatment-by-biomarker interaction test was performed using Cox proportional hazard model. The values of hematologic parameters were categorized using tertile or quartile cutoff points. Variables with a *P*_interaction_ < 0.05 were defined as a potential predictive marker to distinguish the optimal treatment regimen. Subgroup analyses of the biomarkers with *P*_interaction_ < 0.05 were also conducted.

All *P-*values were two-tailed with the significance level set at *P* < 0.05. Analyses were conducted using IBM^®^SPSS^®^ Statistics version 26 and R version 4.0.2. The graphs were plotted using GraphPad Prism version 8.0.

## Results

### Patient Characteristics

Of the 102 patients enrolled in this study, 49 patients received pembrolizumab-pemetrexed-platinum treatment and 57.1% of them received at least 6 doses of pembrolizumab. The remaining 53 patients received bevacizumab-pemetrexed-platinum treatment and 60.4% of them received at least 6 doses of bevacizumab (**Table 1**).

**Table 1:** Baseline patient characteristics.

	Pembrolizumab group (n = 49)	Bevacizumab group (n = 53)	*P*-value
**Age, years, median (IQR)**	64 (59-67)	60 (50-65)	0.005
<65	27 (55.1%)	39 (73.6%)	
≥65	22 (44.9%)	14 (26.4%)	
**Sex**			0.475
male	39 (79.6%)	39 (73.6%)	
female	10 (20.4%)	14 (26.4%)	
**Smoking history**			0.002
Never	12 (24.5%)	29 (54.7%)	
Previous or current	37 (75.5%)	24 (45.3%)	
**ECOG PS**			0.669
0	3 (6.1%)	2 (3.8%)	
1	46 (93.9%)	51 (96.2%)	
**Tumor histology**			0.607
Adenocarcinoma	47 (95.9%)	52 (98.1%)	
Others	2 (4.1%)	1 (1.9%)	
**Stage**			0.182
IIIB~IIIC	4 (8.2%)	9 (17.0%)	
IV	45 (91.8%)	44 (83.0%)	
**Number of metastatic sites**			0.001
0	4 (8.2%)	9 (17.0%)	
1-2	24 (49.0%)	38 (71.7%)	
≥3	21 (42.9%)	6 (11.3%)	
**Metastatic site**			
Bone	25 (51.0%)	16 (30.2%)	0.032
Brain	10 (20.4%)	7 (13.2%)	0.330
Liver	5 (10.2%)	2 (3.8%)	0.256
Chest	40 (81.6%)	38 (71.7%)	0.237
Adrenal gland	13 (26.5%)	7 (13.2%)	0.090
**Hematologic parameters, median (IQR)**			
LDH, U/L	193 (144.50-260.00)	198 (170-229)	0.492
NLR	3.20 (2.35-5.77)	2.49 (1.99-4.21)	0.107
dNLR	2.19 (1.73-3.13)	1.92 (1.49-2.55)	0.098
PLR	149.26 (121.62-196.62)	138.08 (97.13-214.66)	0.177
LMR	2.96 (2.17-3.78)	3.50 (2.30-4.73)	0.252
AEC, ×10^9^/L	0.11 (0.05-0.23)	0.16 (0.09-0.26)	0.181
**PD-L1 TPS**			<0.001
<1%	9 (18.4%)	8 (15.1%)	
1%-49%	9 (18.4%)	1 (1.9%)	
>50%	13 (26.5%)	3 (5.7%)	
unknown	18 (36.7%)	41 (77.4%)	
**Pembrolizumab/Bevacizumab exposure, doses**			0.636
0 to ≤6	21 (42.9%)	21 (39.6%)	
>6 to ≤12	17 (34.7%)	22 (41.5%)	
>12 to ≤18	7 (14.3%)	4 (7.5%)	
>18	4 (8.2%)	6 (11.3%)	
**Radiotherapy combination**			0.118
Yes	10 (20.4%)	5 (9.4%)	
No	39 (79.6%)	48 (90.6%)	

IQR, interquartile range; ECOG PS, Eastern Cooperative Oncology Group performance-status; LDH, lactate dehydrogenase; NLR, neutrophil-to-lymphocyte ratio; dNLR, derived neutrophil-to-lymphocyte ratio; PLR, platelet-to-lymphocyte ratio; LMR, lymphocyte-to-monocyte ratio; AEC, absolute eosinophil count; PD-L1 TPS, programmed death-1 tumor proportion score.

Some percentages may not sum to 100% because of rounding.

The median age was 64 years in the pembrolizumab group and 60 years in the bevacizumab group, with the majority of patients being male and having an ECOG PS score of 1, stage IV adenocarcinoma, common metastatic sites at chest and bone in both groups. A total of 75.5% of patients in the pembrolizumab group and 45.3% of patients in the bevacizumab group were previous or current smokers. A total of 42.9% of patients in the pembrolizumab group had at least 3 metastatic sites compared with 11.3% of patients in the bevacizumab group. Pre-treatment hematologic parameters were also collected, which included LDH, NLR, dNLR, PLR, LMR and AEC. Hematologic data are presented as median (IQR). The detailed characteristics are listed in **Table 1**.

### Efficacy

At the time of the data cutoff, the median duration of follow-up was 8.0 months (IQR, 5.61 to 12.06 months) in the pembrolizumab group versus 16.9 months (IQR, 9.05 to 22.30 months) in the bevacizumab group.

**Figure 1:**
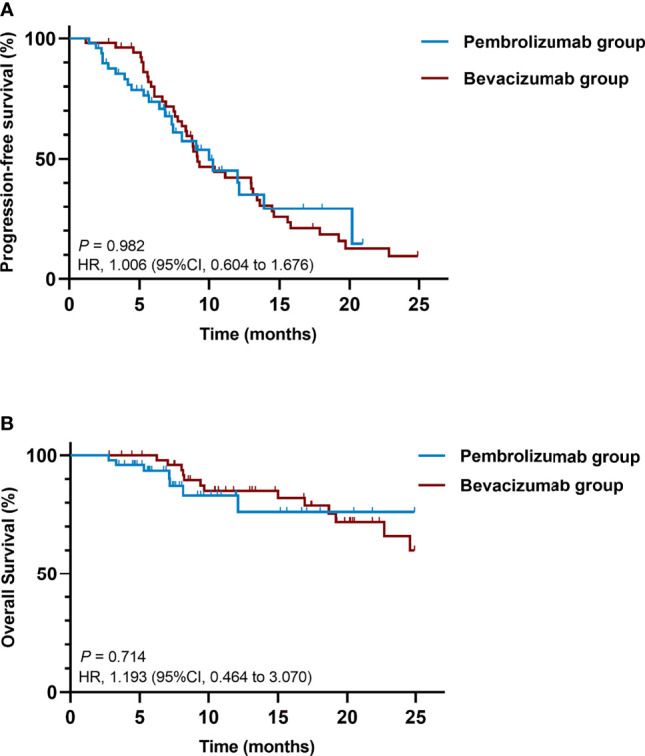
Kaplan-Meier curves of **(A)** PFS and **(B)** OS comparing pembrolizumab-pemetrexed-platinum combination and bevacizumab-pemetrexed-platinum combination. CI, confidence interval; HR, hazard ratio; OS, overall survival; PFS, progression-free survival.

The median PFS was 10.0 months (95% CI, 6.0 to 14.0 months) in the pembrolizumab group and 9.2 months (95% CI, 7.1 to 11.2 months) in the bevacizumab group, with 24 (49.0%) *versus* 41(77.4%) events of disease progression or death, respectively. There was no significant difference in PFS between the two groups (HR = 1.006; 95% CI, 0.604 to 1.676; *P* = 0.982) (**Figure 1A**).

Death occurred in 7 patients (14.3%) in the pembrolizumab group and 13 patients (24.5%) in the bevacizumab group. The median OS was not reached in either group, with no significant difference in OS between the two groups (HR = 1.193; 95% CI, 0.464 to 3.070; *P* = 0.714) (**Figure 1B**).

The ORR was 36.7% (95%CI, 23.8% to 51.2%) *versus* 43.4% (95%CI, 30.1% to 57.7%), and the DCR was 89.8% (95%CI, 77.0% to 96.2%) *versus* 92.5% (95%CI, 80.9% to 97.6%) in the pembrolizumab and bevacizumab group, respectively (**Table 2**). There were no significant differences in ORR and DCR between the two groups. The efficacy analysis in subgroups based on PD-L1 status was not conducted due to the missing data of PD-L1 expression in a large proportion of patients.

**Table 2:** Summary of responses.

	Pembrolizumab group (n = 49)	Bevacizumab group (n = 53)	*P*-value
**Best overall response, n (%)**			
CR	0	0	
PR	18 (36.7)	23 (43.4)	
SD	26 (53.1)	26 (49.1)	
PD	4 (8.2)	3 (5.7)	
No assessment	1 (2.0)	1 (1.9)	
**ORR (95%CI)**	36.7% (23.8% to 51.2%)	43.4% (30.1% to 57.7%)	0.548
**DCR (95%CI)**	89.8% (77.0% to 96.2%)	92.5% (80.9% to 97.6%)	0.735

CR, complete response; PR, partial response; SD stable disease; PD, progressive disease; ORR, objective response rate; DCR, disease control rate.

### Prognostic Biomarkers of Clinical Outcomes

To explore valuable prognostic markers for advanced non-squamous NSCLC patients lacking actionable mutations, we examined the associations between the survival outcomes and the patients’ baseline characteristics.

Univariate analyses of PFS (**Table 3**) and OS (**Table 4**) revealed a negative association between survival outcomes and baseline age (≥65 *vs*. <65 years), tumor histology (others *vs*. adenocarcinoma), bone metastases (yes vs. no), LDH (>300 *vs*. ≤ 300 U/L), NLR (>4.52 *vs*. ≤4.52), dNLR (>2.70 *vs*. ≤2.70), and LMR (≤1.95 *vs*. >1.95).

**Table 3:** Univariable and multivariable analysis of progression-free survival in all patients (n = 102).

Variable	Categorization	Univariable analysis		Multivariable analysis	
		Hazard ratio (95%CI)	*P*-value	Hazard ratio (95%CI)	*P*-value
Treatment	pembrolizumab *vs*. bevacizumab	1.006 (0.604-1.676)	0.982		
Age	≥65 *vs*. <65 years	1.747 (1.060-2.879)	0.029	1.315 (0.742-2.329)	0.348
Sex	male *vs*. female	1.150 (0.644-2.053)	0.637		
Smoking history	yes *vs*. no	1.153 (0.702-1.894)	0.573		
ECOG PS	1 *vs*. 0	0.596 (0.184-1.933)	0.389		
Tumor histology	others *vs*. adenocarcinoma	3.623 (0.842-15.625)	0.087	3.508 (0.696-17.851)	0.128
Stage	IV *vs*. III	1.817 (0.825-4.001)	0.138		
Bone metastases	yes *vs*. no	1.966 (1.174-3.291)	0.010	1.590 (0.879-2.874)	0.125
Brain metastases	yes *vs*. no	1.587 (0.844-2.984)	0.152		
Liver metastases	yes *vs*. no	0.655 (0.237-1.808)	0.414		
Chest metastases	yes *vs*. no	1.289 (0.722-2.302)	0.390		
Adrenal gland metastases	yes *vs*. no	1.278 (0.703-2.325)	0.421		
LDH	>300 *vs*. ≤300 U/L	2.819 (1.374-5.782)	0.005	2.034 (0.900-4.595)	0.088
NLR	>4.52 *vs*. ≤4.52	2.108 (1.219-3.646)	0.008	1.323 (0.442-3.960)	0.617
dNLR	>2.70 *vs*. ≤2.70	1.757 (0.978-3.156)	0.060	0.698 (0.257-1.895)	0.480
PLR	>118.34 *vs*. ≤118.34	1.273 (0.760-2.135)	0.359		
**LMR**	**>1.95 *vs*. ≤1.95**	**0.353 (0.193-0.643)**	**0.001**	**0.312 (0.171-0.570)**	**<0.001**
AEC	>0.19 *vs*. ≤0.19 ×10^9^/L	0.701 (0.416-1.180)	0.181		
Radiotherapy combination	yes *vs*. no	1.354 (0.701-2.614)	0.367		

ECOG PS, Eastern Cooperative Oncology Group performance-status; LDH, lactate dehydrogenase; NLR, neutrophil-to-lymphocyte ratio; dNLR, derived neutrophil-to-lymphocyte ratio; PLR, platelet-to-lymphocyte ratio; LMR, lymphocyte-to-monocyte ratio; AEC, absolute eosinophil count. Statistically significant values are bolded.

**Table 4:** Univariable and multivariable analysis of overall survival in all patients (n = 102).

Variable	Categorization	Univariable analysis		Multivariable analysis	
		Hazard ratio (95%CI)	*P*-value	Hazard ratio (95%CI)	*P*-value
Treatment	pembrolizumab *vs.* bevacizumab	1.193 (0.464-3.070)	0.714		
Age	≥65 *vs.* <65 years	1.265 (0.953-2.380)	0.079	1.222 (0.395-3.779)	0.728
Sex	male *vs.* female	0.905 (0.325-2.519)	0.848		
Smoking history	yes *vs.* no	1.242 (0.507-3.044)	0.636		
ECOG	1 *vs.* 0	0.960 (0.350-2.634)	0.937		
Tumor histology	others *vs.* adenocarcinoma	4.073 (0.523-31.370)	0.180		
Stage	IV *vs.* III	3.142 (0.419-23.550)	0.265		
**Bone metastases**	**yes *vs.* no**	**5.361 (1.998-14.381)**	**0.001**	**4.107 (1.434-11.761)**	**0.009**
Brain metastases	yes *vs.* no	1.433 (0.464-4.424)	0.531		
Liver metastases	yes *vs.* no	0.043 (0.001-68.446)	0.404		
Chest metastases	yes *vs.* no	1.913 (0.557-6.575)	0.303		
Adrenal gland metastases	yes *vs.* no	1.376 (0.455-4.160)	0.572		
**LDH**	**>300 *vs.* ≤300 U/L**	**5.871 (2.165-15.925)**	**0.001**	**4.300 (1.206-15.330)**	**0.025**
NLR	>4.52*vs.* ≤4.52	4.134 (1.652-10.349)	0.002	1.626 (0.376-7.030)	0.516
dNLR	>2.70 *vs.* ≤2.70	2.238 (0.841-5.956)	0.107	0.359 (0.085-1.523)	0.165
PLR	>118.34 *vs.* ≤118.34	1.941 (0.700-5.382)	0.203		
**LMR**	**>1.95 *vs.* ≤1.95**	**0.120 (0.044-0.326)**	**<0.001**	**0.189 (0.068-0.523)**	**0.039**
AEC	>0.19 *vs.* ≤0.19 × 10^9^/L	0.701 (0.416-1.180)	0.181		
Radiotherapy combination	yes *vs.* no	0.921 (0.496-1.710_	0.794		

ECOG PS, Eastern Cooperative Oncology Group performance-status; LDH, lactate dehydrogenase; NLR, neutrophil-to-lymphocyte ratio; dNLR, derived neutrophil-to-lymphocyte ratio; PLR, platelet-to-lymphocyte ratio; LMR, lymphocyte-to-monocyte ratio; AEC, absolute eosinophil count. Statistically significant values are bolded.

Further multivariate analyses confirmed that higher baseline LMR (>1.95 *vs*. ≤1.95, HR = 0.312, 95%CI [0.171-0.570], *P* < 0.001) was an independent prognostic factor of longer PFS. Presence of baseline bone metastasis (yes *vs*. no, HR = 4.107, 95% CI [1.434-11.761], *P* = 0.009) and higher baseline LDH (>300 *vs*. ≤300 U/L HR = 4.300, 95% CI [1.206-15.330], *P* = 0.025) were independent indicators for poorer OS. Elevated baseline LMR (>1.95 *vs*. ≤1.95, HR = 0.189, 95% CI [0.068-0.523], *P* = 0.039) was an independent prognostic factor of prolonged OS. The detailed results of the univariable and multivariable analyses are listed in **Tables 3**, **4**.

### Predictive Biomarkers and Subgroup Analysis

Next, we performed the analysis of treatment-by-biomarker interactions to evaluate the candidate biomarkers for the prediction of treatment effect difference between the pembrolizumab group and bevacizumab group.

**Figure 2:**
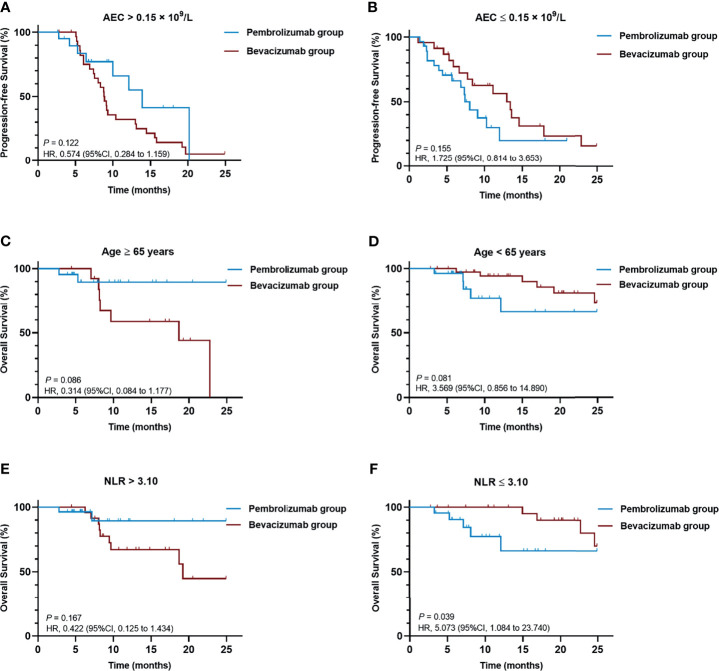
Kaplan-Meier curves of **(A)** PFS of patients with baseline AEC > 0.15 × 10^9^/L, **(B)** PFS of patients with baseline AEC ≤ 0.15 × 10^9^/L, **(C)** OS of patients aged ≥ 65 years, **(D)** OS of patients aged < 65 years, **(E)** OS of patients with baseline NLR >3.10, **(F)** OS of patients with baseline NLR ≤ 3.10. AEC, absolute eosinophil count; CI, confidence interval; HR, hazard ratio; NLR, neutrophil-to-lymphocyte ratio; OS, overall survival; PFS, progression-free survival.

In the statistical treatment-by-biomarker interaction test related to PFS, baseline AEC at a cutoff point of 0.15 × 10^9/^L, *P* = 0.027) was detected to be a potential predictive biomarker (**Supplementary Table 1**). In the AEC-biomarker-positive group (AEC > 0.15 × 10^9^/L), the pembrolizumab combination showed longer PFS than the bevacizumab combination (HR = 0.574, 95%CI [0.284 to 1.159], *P* = 0.122), while the latter one outperformed in the AEC-biomarker-negative (AEC ≤ 0.15 × 10^9^/L) patients (HR = 1.725, 95%CI [0.814 to 3.653], *P* = 0.155), although no significant difference was observed in either case (**Figures 2A, B**).

For biomarkers related to better OS, age (≥65 *vs*. <65 years, *P* = 0.019) and baseline NLR (>3.10 *vs*. ≤3.10, *P* = 0.025) were detected to be of statistical significance in the treatment-by-biomarker interaction test (**Supplementary Table 2**). Patients over 65 years of age appeared to benefit more from the pembrolizumab combination treatment (HR = 0.314, 95%CI [0.084 to 1.177], *P* = 0.086), while the treatment of bevacizumab combination was superior for the OS of patients under the age of 65 years (HR = 3.569, 95%CI [0.856 to 14.890], *P* = 0.081), though no significant difference in either case was seen (**Figures 2C, D**). In NLR-biomarker-positive (NLR >3.10) group, the pembrolizumab combination tended to demonstrate more OS benefits but the difference was not significant (HR = 0.422, 95%CI [0.125 to 1.434], P = 0.167). For patients with baseline NLR ≤3.10, the bevacizumab combination elicited significantly prolonged OS (HR = 5.073, 95%CI [1.084 to 23.740], P = 0.039) (**Figures 2E, F**).

### Safety

AEs of any grade occurred in 46 patients (93.9%) in the pembrolizumab group and 50 patients (94.3%) in the bevacizumab group (**Table 5**). AEs of grade 3 or worse were noted in 25 patients (51.0%) and 17 patients (32.1%), respectively. Among patients in the pembrolizumab group, the most common AEs were leukopenia (40.8%), anemia (34.7%), and fatigue (30.6%). In the bevacizumab group, the most common AEs were anemia (54.7%), elevated aminotransferase (47.2%), and abnormal coagulation parameters (45.3%).

**Table 5:** Overview of treatment-related adverse events.

	Pembrolizumab group (n = 49)		Bevacizumab group (n = 53)	
	Any grade	Grade 3-5	Any grade	Grade 3-5
**Any event, n (%)**	46 (93.9)	25 (51.0)	50 (94.3)	17 (32.1)
**Event related to treatment discontinuation, n (%)**	1 (2.0)	1 (2.0)	2 (3.8)	1 (1.9)
**Event related to death, n (%)**	1 (2.0)	1 (2.0)	0	0
**Event occurring in > 5% of patients in either group, n (%)**				
Leukopenia	20 (40.8)	6 (12.2)	15 (28.3)	2 (3.8)
Anemia	17 (34.7)	5 (10.2)	29 (54.7)	3 (5.7)
Fatigue	15 (30.6)	0	13 (24.5)	0
Neutropenia	13 (26.5)	6 (12.2)	19 (35.8)	1 (1.9)
Thrombocytopenia	12 (24.5)	4 (8.2)	17 (32.1)	5 (9.4)
Elevated aminotransferase	10 (20.4)	1 (2.0)	25 (47.2)	1 (1.9)
Pyrexia	7 (14.3)	0	15 (28.3)	0
Rash	6 (12.2)	0	7 (13.2)	0
Abnormal coagulation parameters	6 (12.2)	0	24 (45.3)	2 (3.8)
Constipation	6 (12.2)	0	9 (17.0)	0
Pneumonitis	5 (10.2)	1 (2.0)	0	0
Nausea	4 (8.2)	1 (2.0)	2 (3.8)	0
Elevated blood creatinine	3 (6.1)	0	10 (18.9)	0
Vomiting	3 (6.1)	0	2 (3.8)	0
Hypothyroidism	3 (6.1)	0	0	0
Abnormal electrocardiogram	1 (2.0)	0	9 (17.0)	1 (1.9)
Proteinuria	1 (2.0)	0	7 (13.2)	1 (1.9)
Hemorrhage	1 (2.0)	0	13 (24.5)	2 (3.8)
Hypertension	0	0	8 (15.1)	1 (1.9)

The events are listed in descending order of frequency in the pembrolizumab group.

The most frequently occurring immune-mediated AEs in the pembrolizumab group were pneumonitis (10.2%) and hypothyroidism (6.1%). Bevacizumab-mediated AEs such as hemorrhage (24.5%), hypertension (15.1%), and proteinuria (13.2%) were frequently observed in the bevacizumab group.

The causes of AEs-related treatment discontinuation were reported as severe thrombocytopenia (n=1) in the pembrolizumab group, hemorrhage (n=1), and thromboembolism (n=1) in the bevacizumab group. There was one death in the pembrolizumab group due to severe immune-mediated pneumonia, and no AE-related death in the bevacizumab group.

## Discussion

To the best of our knowledge, we performed the first real-world study comprehensively comparing pembrolizumab versus bevacizumab in combination with pemetrexed-platinum as first-line treatment in driver-gene wild-type advanced non-squamous NSCLC. Our retrospective analysis showed no statistically significant differences in PFS, OS, ORR, or DCR between the pembrolizumab and bevacizumab groups in this population of patients.

In the final analysis of the large, open-label, phase III IMpower150 study (19, 20), numerically but not statistically significant OS improvement was shown in atezolizumab (anti-PD-L1 antibody) -carboplatin-paclitaxel treatment compared with bevacizumab-carboplatin-paclitaxel treatment (median OS, 19.0 vs. 15.0 months; HR=0.86; 95%CI [0.73 to 1.01]) for metastatic non-squamous NSCLC patients, regardless of PD-L1 level, EGFR/ALK genetic alterations or baseline liver metastasis status. Consistent with the corresponding results in the IMpower150 study, the results of the current study found that the addition of pembrolizumab or bevacizumab to first-line standard chemotherapy generally conferred similar levels of clinical outcome improvements to patients.

The presence of systemic inflammation is closely associated with lower therapeutic response and worse prognosis in tumor patients (16), therefore, it has been extensively investigated for tumor biomarker discovery. Elevated LDH level is correlated with high tumor burden and is considered as a prognostic marker for worse survival in advanced NSCLC patients receiving immunotherapy (21, 22). Neutrophils have been shown to not only suppress antitumor immune responses *via* generating some chemokines and cytokines (23), but also promote tumor progression through stimulating angiogenesis by releasing pro-angiogenic factors such as VEGF (24). Lymphocytes such as CD8+ T cells are crucial to antitumor immunity (25), and activated eosinophils could enhance the infiltration of CD8+ T cells within the tumor microenvironment (26). Platelets play an active role in the inflammatory process and have been found to be involved in tumor metastasis through their interactions with tumor cells (27). Peripheral monocytes are reported to promote tumor development and suppress immunosurveillance (28). Accordingly, other studies have indicated that high NLR, high dNLR, high PLR, low LMR, and low AEC at baseline were significantly correlated with inferior survival outcomes in advanced NSCLC patients treated with immunotherapy or chemotherapy alone (15–17, 29, 30). Here we checked the chemotherapy-naive, advanced, non-squamous NSCLC patients carrying no targetable EGFR, ALK or ROS1 genetic mutations regardless of their PD-L1 levels. Pre-treatment LDH >300 U/L appeared to be an independent factor for worse OS, and baseline LMR >1.95 appeared to be a prognostic biomarker for both significantly prolonged PFS and OS. We did not observe the prognostic role of baseline NLR, dNLR, PLR, or AEC in our study, which may be due to the small sample size.

Bone metastasis was a poor prognostic factor for unfavorable OS in the overall patient population of this study. This could be attributed to a higher risk of skeletal-related events such as chronic bone pain, spinal cord or nerve root compression, hypercalcemia, and pathological fractures (31), leading to the deterioration of life quality as well as the impaired function of bone marrow in regulating immune system (32). A pooled analysis of two phase I/II trials showed that the introduction of radiotherapy could improve the clinical outcomes in metastatic NSCLC patients treated with pembrolizumab (33). However, the efficacy of adding radiotherapy to bevacizumab combined with chemotherapy in advanced NSCLC patients remained controversial, as the relevant studies were terminated early due to the high incidences of severe AEs (34, 35). In our study, patients who received radiotherapy tended to be at stage IIIB~ IIIC or had oligometastatic disease with a relatively low tumor burden (36), and we did not observe the positive prognostic effect of radiotherapy regarding PFS or OS in the overall study population. Further studies with more subjects are needed to determine the role of radiotherapy to verify our results.

Patient stratification for the selection of optimal treatment is critical. Pre-treatment NLR ≤3.10 was observed to be a predictive biomarker indicating significantly better OS outcomes in the bevacizumab-pemetrexed-platinum treatment compared with pembrolizumab-pemetrexed-platinum treatment. High baseline NLR was reported to associate with poor prognosis in advanced NSCLC patients receiving bevacizumab or PD-1 inhibitors (16, 37, 38). Angiogenesis inhibitor was found to increase T lymphocyte infiltration into tumors and promote dendritic cell maturation (39), while neutrophils were found to promote angiogenesis by releasing different pro-angiogenic factors that are potentially independent of VEGF (37). Currently, there are no studies comparing the efficacies of immune checkpoint inhibitors and angiogenesis inhibitors in NSCLC patients with low baseline NLR condition. The mechanisms underlying the interactions between the peripheral inflammatory cells and bevacizumab response remain obscure. This finding in our study requires further investigation.

Numerically better PFS and OS were observed in subgroup analyses of patients receiving the immunotherapy combination in populations stratified by the markers including baseline AEC > 0.15 × 10^9^/L, age ≥ 65 years and baseline NLR > 3.10. However, there were no significant benefits of the immunotherapy combination in the above patient stratifications, which may be owing to the limited number of samples. Moreover, the higher proportion of multiple metastatic sites (≥3) in the pembrolizumab group may result in poor responses and impair the survival of patients in the pembrolizumab group (40). Collectively, the above factors may help clinicians select the appropriate first-line treatment for individual patients.

Although the proportion of Grade 3 and worse AEs was higher in the pembrolizumab group than in the bevacizumab group, both combination chemotherapies showed acceptable toxicities. The frequencies of treatment-related AEs in the two groups were essentially similar to those reported in previous pembrolizumab-chemotherapy or bevacizumab-chemotherapy combination studies (4, 5, 7, 41). Furthermore, no new safety concerns were raised in either group during this study.

We acknowledge several limitations of this study, including the retrospective single-center study design, relatively small sample size, and immature OS data. Although the univariate and multivariate analyses of biomarkers related to overall survival require further clinical information, our results could still be a reference material for future similar studies. Secondly, the imbalance in age, smoking histories, and the number of metastatic foci between the two groups could be confounding factors and may partially explain the relatively poorer physical conditions among patients in the pembrolizumab group. Also, because the PD-L1 status was unavailable for most patients receiving the bevacizumab combination therapy, we could not directly compare the PD-L1 levels between the two groups to statistically evaluate the treatment efficacy. Thus, further follow-up of the two groups and a randomized, prospective control study with a larger sample cohort are needed to verify our results. Additionally, we were unable to investigate the effects of steroids in the pembrolizumab treatment cohort, because both groups received standard steroid pre-medication before pemetrexed administration, and the patients with brain metastasis were asymptomatic and not administered with steroids. Although the use of steroids is associated with poor outcomes in NSCLC patients treated with PD-1 axis inhibitors, several clinical trials have suggested that the addition of pembrolizumab can benefit patients despite the administration of steroids (42).

Collectively, we investigated the combined treatment of the immune checkpoint inhibitor, pembrolizumab, or the angiogenesis inhibitor, bevacizumab, with pemetrexed-platinum herein. Comparable efficacies and safety in chemotherapy-naive, advanced, non-squamous NSCLC patients lacking driver mutations were identified for both treatments. We also discovered useful prognostic and predictive biomarkers that can be used to identify patients who can derive the maximum benefits from such therapies. Therefore, our findings may aid clinical decision-making for optimal therapeutic regimens in front-line treatment of advanced non-squamous NSCLC, and provide insights that will advance future prospective trials.

## Data Availability Statement

The original contributions presented in the study are included in the article/**Supplementary Material**. Further inquiries can be directed to the corresponding authors.

## Ethics Statement

The studies involving human participants were reviewed and approved by Fudan University Shanghai Cancer Center. The patients/participants provided their written informed consent to participate in this study.

## Author Contributions

JW and XZ conceived and designed the study. JL and CL wrote the manuscript. JL, CL and QL collected and analyzed the patient data. XW, HW, HY, SS, YZ and YL were involved in the patient management and provided the study materials. JW and XZ verified the integrity of the data and edited the manuscript. All authors contributed to the article and approved the submitted version.

## Funding

This study was sponsored by the Natural Science Foundation of Shanghai (grant number: 19ZR1410400) and Shanghai Municipal Health Commission (grant number: 2020CXJQ02).

## Conflict of Interest

The authors declare that the research was conducted in the absence of any commercial or financial relationships that could be construed as a potential conflict of interest.

## Publisher’s Note

All claims expressed in this article are solely those of the authors and do not necessarily represent those of their affiliated organizations, or those of the publisher, the editors and the reviewers. Any product that may be evaluated in this article, or claim that may be made by its manufacturer, is not guaranteed or endorsed by the publisher.
