# Supplementary material for: Direct Comparison Between the Addition of Pembrolizumab or Bevacizumab for Chemotherapy-Based First-Line Treatment of Advanced Non-Squamous Non-Small Cell Lung Cancer Lacking Driver Mutations
Source: Front Oncol. 2021 Sep 29;11:752545. doi: 10.3389/fonc.2021.752545 (PMC8511673; doi:10.3389/fonc.2021.752545)
Supplement: Supplementary file 1 [file Table_1.docx]

**Supplementary Table 1. Interaction analyses of treatment with candidate biomarkers for progression-free survival in Cox proportional hazards model.**

| **Biomarker** | **Categorization** | **Coefficient** | **Hazard ratio** | **Standard error** | **Wald Z value** | **P value** |
| --- | --- | --- | --- | --- | --- | --- |
| Age | ≥65 vs. <65 years | -0.840 | 0.432 | 0.550 | -1.527 | 0.127 |
| Sex | male vs. female | 0.381 | 1.463 | 0.608 | 0.626 | 0.532 |
| Smoking history | yes vs. no | 0.002 | 1.002 | 0.570 | 0.004 | 0.997 |
| ECOG | 1 vs. 0 | -1.922 | 0.146 | 1.311 | -1.466 | 0.143 |
| Tumor histology | others vs. adenocarcinoma | 0.873 | 2.395 | 1.441 | 0.606 | 0.544 |
| Stage | Ⅳ vs. Ⅲ | -0.240 | 0.787 | 1.117 | -0.214 | 0.830 |
| Bone metastases | yes vs. no | -0.142 | 0.868 | 0.538 | -0.263 | 0.792 |
| Brain metastases | yes vs. no | -0.350 | 0.705 | 0.649 | -0.539 | 0.590 |
| Liver metastases | yes vs. no | 1.252 | 3.497 | 1.038 | 1.206 | 0.228 |
| Chest metastases | yes vs. no | 0.191 | 1.210 | 0.621 | 0.307 | 0.759 |
| Adrenal gland metastases | yes vs. no | 0.237 | 1.267 | 0.627 | 0.378 | 0.706 |
| Radiotherapy combination | yes vs. no | -0.643 | 0.526 | 0.678 | -0.949 | 0.342 |
| LDH | ＞153.00 vs. ≤153.00 U/L | -0.113 | 0.893 | 0.653 | -0.173 | 0.863 |
| LDH | ＞172.00 vs. ≤172.00 U/L | -0.957 | 0.384 | 0.635 | -1.507 | 0.132 |
| LDH | ＞195.00 vs. ≤195.00 U/L | -0.137 | 0.872 | 0.521 | -0.263 | 0.793 |
| LDH | ＞221.33 vs. ≤221.33 U/L | -0.309 | 0.734 | 0.558 | -0.554 | 0.579 |
| LDH | ＞243.50 vs. ≤243.50 U/L | -0.954 | 0.385 | 0.596 | -1.600 | 0.109 |
| NLR | ＞2.13 vs. ≤2.13 | 0.081 | 1.085 | 0.647 | 0.126 | 0.900 |
| NLR | ＞2.32 vs. ≤2.32 | -0.643 | 0.526 | 0.634 | -1.014 | 0.311 |
| NLR | ＞3.10 vs. ≤3.10 | 0.641 | 1.899 | 0.521 | 1.230 | 0.219 |
| NLR | ＞3.78 vs. ≤3.78 | -0.119 | 0.887 | 0.535 | -0.223 | 0.823 |
| NLR | ＞4.87 vs. ≤4.87 | -0.759 | 0.468 | 0.581 | -1.305 | 0.192 |
| dNLR | ＞1.57 vs. ≤1.57 | -0.113 | 0.893 | 0.653 | -0.173 | 0.863 |
| dNLR | ＞1.77 vs. ≤1.77 | -0.957 | 0.384 | 0.635 | -1.507 | 0.132 |
| dNLR | ＞2.10 vs. ≤2.10 | -0.137 | 0.872 | 0.521 | -0.263 | 0.793 |
| dNLR | ＞2.55 vs. ≤2.55 | -0.309 | 0.734 | 0.558 | -0.554 | 0.579 |
| dNLR | ＞2.67 vs. ≤2.67 | -0.954 | 0.385 | 0.596 | -1.600 | 0.109 |
| PLR | ＞110.82 vs. ≤110.82 | 0.623 | 1.864 | 0.644 | 0.966 | 0.334 |
| PLR | ＞122.13 vs. ≤122.13 | 0.054 | 1.055 | 0.568 | 0.095 | 0.925 |
| PLR | ＞141.48 vs. ≤141.48 | -0.591 | 0.554 | 0.523 | -1.131 | 0.258 |
| PLR | ＞181.16 vs. ≤181.16 | -0.589 | 0.555 | 0.542 | -1.087 | 0.277 |
| PLR | ＞201.56 vs. ≤201.56 | -0.349 | 0.706 | 0.626 | -0.557 | 0.577 |
| LMR | ＞2.26 vs. ≤2.26 | 0.299 | 1.348 | 0.577 | 0.517 | 0.605 |
| LMR | ＞2.63 vs. ≤2.63 | -0.063 | 0.939 | 0.542 | -0.116 | 0.908 |
| LMR | ＞3.08 vs. ≤3.08 | -0.732 | 0.481 | 0.537 | -1.364 | 0.172 |
| LMR | ＞3.94 vs. ≤3.94 | -0.574 | 0.563 | 0.584 | -0.983 | 0.326 |
| LMR | ＞4.40 vs. ≤4.40 | -0.613 | 0.542 | 0.625 | -0.980 | 0.327 |
| AEC | ＞0.08 vs. ≤0.08 ×10^9^/L | 0.451 | 1.569 | 0.592 | 0.761 | 0.447 |
| AEC | ＞0.09 vs. ≤0.09 ×10^9^/L | 0.294 | 1.341 | 0.568 | 0.517 | 0.605 |
| **AEC** | **＞0.15 vs. ≤0.15 ×10^9^/L** | **1.202** | **3.327** | **0.545** | **2.205** | **0.027** |
| AEC | ＞0.21 vs. ≤0.21 ×10^9^/L | 0.887 | 2.429 | 0.600 | 1.479 | 0.139 |
| AEC | ＞0.24 vs. ≤0.24 ×10^9^/L | 0.487 | 1.627 | 0.610 | 0.799 | 0.425 |

**Supplementary Table 2. Interaction analyses of treatment with candidate biomarkers for overall survival in Cox proportional hazards model.**

| **Biomarker** | **Categorization** | **Coefficient** | **Hazard ratio** | **Standard error** | **Wald Z value** | **P value** |
| --- | --- | --- | --- | --- | --- | --- |
| **Age** | **≥65 vs. <65 years** | **2.379** | **10.793** | **1.018** | **2.336** | **0.019** |
| Sex | male vs. female | 1.389 | 4.012 | 1.091 | 1.273 | 0.203 |
| Smoking history | yes vs. no | 0.121 | 1.128 | 1.010 | 0.120 | 0.905 |
| ECOG | 1 vs. 0 | -15.797 | 0.000 | 4693.167 | -0.003 | 0.997 |
| Tumor histology | others vs. adenocarcinoma | 17.754 | 51358729.230 | 7767.521 | 0.002 | 0.998 |
| Stage | Ⅳ vs. Ⅲ | -16.229 | 0.000 | 7663.301 | -0.002 | 0.998 |
| Bone metastases | yes vs. no | -1.083 | 0.338 | 1.218 | -0.889 | 0.374 |
| Brain metastases | yes vs. no | 1.228 | 3.416 | 1.266 | 0.970 | 0.332 |
| Liver metastases | yes vs. no | 0.331 | 1.392 | 14670.936 | 0.000 | 1.000 |
| Chest metastases | yes vs. no | 0.262 | 1.300 | 1.327 | 0.197 | 0.843 |
| Adrenal gland metastases | yes vs. no | 0.020 | 1.021 | 1.140 | 0.018 | 0.986 |
| Radiotherapy combination | yes vs. no | -1.504 | 0.222 | 1.355 | -1.110 | 0.267 |
| LDH | ＞153.00 vs. ≤153.00 U/L | 17.127 | 27431409.320 | 5108.376 | 0.003 | 0.997 |
| LDH | ＞172.00 vs. ≤172.00 U/L | 0.981 | 2.666 | 1.338 | 0.733 | 0.464 |
| LDH | ＞195.00 vs. ≤195.00 U/L | 0.513 | 1.671 | 0.976 | 0.526 | 0.599 |
| LDH | ＞221.33 vs. ≤221.33 U/L | 0.507 | 1.661 | 0.948 | 0.535 | 0.593 |
| LDH | ＞243.50 vs. ≤243.50 U/L | 0.497 | 1.644 | 0.964 | 0.516 | 0.606 |
| NLR | ＞2.13 vs. ≤2.13 | 1.766 | 5.848 | 1.507 | 1.172 | 0.241 |
| NLR | ＞2.32 vs. ≤2.32 | 0.402 | 1.494 | 1.266 | 0.317 | 0.751 |
| **NLR** | **＞3.10 vs. ≤3.10** | **2.328** | **10.255** | **1.039** | **2.241** | **0.025** |
| NLR | ＞3.78 vs. ≤3.78 | 1.573 | 4.821 | 1.012 | 1.554 | 0.120 |
| NLR | ＞4.87 vs. ≤4.87 | 1.425 | 4.157 | 1.015 | 1.404 | 0.160 |
| dNLR | ＞1.57 vs. ≤1.57 | 19.267 | 233154988.700 | 7176.058 | 0.003 | 0.998 |
| dNLR | ＞1.77 vs. ≤1.77 | -0.304 | 0.738 | 1.237 | -0.246 | 0.806 |
| dNLR | ＞2.10 vs. ≤2.10 | 1.849 | 6.356 | 0.979 | 1.889 | 0.059 |
| dNLR | ＞2.55 vs. ≤2.55 | 1.484 | 4.410 | 1.009 | 1.470 | 0.142 |
| dNLR | ＞2.67 vs. ≤2.67 | 1.335 | 3.802 | 1.224 | 1.091 | 0.275 |
| PLR | ＞110.82 vs. ≤110.82 | 1.025 | 2.787 | 1.273 | 0.805 | 0.421 |
| PLR | ＞122.13 vs. ≤122.13 | 1.272 | 3.567 | 1.067 | 1.192 | 0.233 |
| PLR | ＞141.48 vs. ≤141.48 | 0.467 | 1.595 | 0.957 | 0.488 | 0.626 |
| PLR | ＞181.16 vs. ≤181.16 | -0.319 | 0.727 | 0.956 | -0.333 | 0.739 |
| PLR | ＞201.56 vs. ≤201.56 | -0.026 | 0.975 | 1.017 | -0.025 | 0.980 |
| LMR | ＞2.26 vs. ≤2.26 | -1.136 | 0.321 | 0.956 | -1.189 | 0.234 |
| LMR | ＞2.63 vs. ≤2.63 | -0.611 | 0.543 | 0.981 | -0.622 | 0.534 |
| LMR | ＞3.08 vs. ≤3.08 | -1.624 | 0.197 | 0.975 | -1.665 | 0.096 |
| LMR | ＞3.94 vs. ≤3.94 | -1.209 | 0.299 | 1.073 | -1.127 | 0.260 |
| LMR | ＞4.40 vs. ≤4.40 | -0.749 | 0.473 | 1.332 | -0.562 | 0.574 |
| AEC | ＞0.08 vs. ≤0.08 ×10^9^/L | -0.590 | 0.554 | 1.329 | -0.444 | 0.657 |
| AEC | ＞0.09 vs. ≤0.09 ×10^9^/L | -1.000 | 0.368 | 1.267 | -0.789 | 0.430 |
| AEC | ＞0.15 vs. ≤0.15 ×10^9^/L | 0.045 | 1.046 | 0.973 | 0.046 | 0.963 |
| AEC | ＞0.21 vs. ≤0.21 ×10^9^/L | -0.684 | 0.505 | 0.954 | -0.717 | 0.473 |
| AEC | ＞0.24 vs. ≤0.24 ×10^9^/L | -0.929 | 0.395 | 0.973 | -0.955 | 0.340 |
